# Social Representations of Hesitant Brazilians about Vaccination against COVID-19

**DOI:** 10.3390/ijerph20136204

**Published:** 2023-06-22

**Authors:** Keila Cristina Oliveira dos Santos, Maria de Fátima Junqueira-Marinho, Adriana Teixeira Reis, Karla Gonçalves Camacho, Marcio Fernandes Nehab, Dimitri Marques Abramov, Zina Maria Almeida de Azevedo, Livia Almeida de Menezes, Margarida dos Santos Salú, Carlos Eduardo da Silva Figueiredo, Maria Elisabeth Lopes Moreira, Zilton Farias Meira de Vasconcelos, Flavia Amendola Anisio de Carvalho, Livia de Rezende de Mello, Roberta Fernandes Correia, Saint Clair dos Santos Gomes Junior, Daniella Campelo Batalha Cox Moore

**Affiliations:** 1 National Institute of Women, Children and Adolescents Health Fernandes Figueira (IFF-FIOCRUZ), Rio de Janeiro 22250-020, Brazil; 2 Institute of Child Care and Pediatrics Martagao Gesteira, Federal University of Rio de Janeiro (UFRJ), Rio de Janeiro 21941-912, Brazil; 3 State of Rio de Janeiro University, Pedro Ernesto Hospital, Rio de Janeiro 20551-030, Brazil; 4 School of Medicine Unigranrio, University of Grande Rio, Duque de Caxias 25071-202, Brazil; 5 School of Medicine, Internal Medicine (UFF), Fluminense Federal University, Niterói 24020-000, Brazil

**Keywords:** COVID-19, vaccine, COVID-19 vaccines, vaccine hesitancy, hesitancy determinants, Brazil

## Abstract

Background: The control of the COVID-19 pandemic has been a great challenge. Understanding the thoughts and beliefs underlying vaccine hesitancy can help in the formulation of public policies. The present study aimed to analyze the social representations of hesitant Brazilians about vaccination against COVID-19. Methods: Qualitative research guided by the Theory of Social Representations, carried out through an online survey among Brazilian adults living in Brazil. The data were analyzed using the IRaMuTeQ software. Results: Of the 173,178 respondents, 10,928 were hesitant and declared reasons for vaccination hesitation. The analysis generated three classes: mistrust of the vaccine and underestimation of the severity of the pandemic; (dis)information and distrust of political involvement; and fear of adverse reactions to COVID-19 vaccines. Conclusions: Social knowledge, presented by the representations apprehended in this study, demonstrates difficulty in discerning the reliability of information and a social imagination full of doubts and uncertainties. Understanding the internal dynamics of these groups, with their representations of the world, is important to propose policies and actions that echo and cause changes in the understanding of the role of immunization. It is essential to shed light on the sociological imagination so that gaps filled with false information can be dismantled and confronted with scientific knowledge accessible to the population.

## 1. Introduction

The continued resurgence of vaccine-preventable diseases led the WHO to name vaccine hesitancy as one of the top ten global health threats in 2019. Anti-vaccine content, in addition to hesitancy in the long-known vaccines, further threatens the uptake of emerging vaccines, such as the COVID-19 vaccine [1].

Vaccines are some of the most successful public health initiatives ever implemented. Vaccination is one of the most economical ways to avoid diseases: currently it avoids 2 to 3 million deaths per year and more than 1.5 million could be avoided if global vaccination coverage improved. Despite that, vaccine hesitancy, which is defined as reluctance or refusal to vaccinate despite the availability of vaccines, is still a challenge. This indecision to vaccinate causes a setback in the fight against vaccine-preventable diseases [2].

Despite the national political context, the current anti-vaccine movements and the proliferation of controversial opinions on social networks, it was observed that 89.5% of Brazilians had an intention to vaccinate [3].

Confidence in the vaccine is a matter of paramount importance that directly impacts global public health. The decrease in confidence leaves countries more vulnerable to disease outbreaks and rewinds from eradicated diseases. There is research showing a clear association between lower levels of vaccine confidence and higher levels of vaccine hesitancy [4]. In 2019, the World Health Organization declared vaccine hesitancy 1 of the 10 global health threats, in part due to outbreaks of vaccine-preventable diseases, such as measles and diphtheria [5]. In that same year, Brazil lost the measles eradication certificate, showing that vaccine hesitancy was already a public health problem for us even before the COVID-19 pandemic arrived, since the measles vaccine is available for our children free of charge in the Brazilian public health system.

This study aimed to analyze the social representations about vaccine hesitancy against COVID-19 among Brazilians in order to enable the construction of public policies that help improve vaccine adherence.

## 2. Materials and Methods

### 2.1. Recruitment and Study Design

This is a qualitative study, guided by the Theory of Social Representations (TRS). Through the TRS, we can better understand social phenomena from the knowledge of ideologies and certain behaviors. Thus, the representations about a particular object or phenomenon are worldviews impregnated with sociocultural values, which are built along a life trajectory. The focus of the TRS is the understanding of beliefs and values of social groups that can impact on social health practices. 

The recruitment of the study was voluntary, anonymous, online, linked to the research entitled “Evaluation of Brazilians’ intention to vaccinate for prevention of COVID-19” and conducted from 22–29 January 2021, through a free platform google forms with the link made available on social networks WhatsApp, Telegram, Facebook, Instagram, Twitter and LinkedIn. The inclusion criteria for the analysis carried out in this study were being Brazilian, being over 18 years old and having answered the following question: “Why are you in doubt or do you intend not to vaccinate against COVID-19?”, which was made available to the vaccine-hesitant people (those who responded that they did not intend to vaccinate, were in doubt or could delay their vaccination by preference to one given vaccine to the detriment of another. The exclusion criterion was to have used the response field for comments that did not correspond to reasons of vaccine hesitancy.

### 2.2. Data Processing and Analysis

Software was used as a tool for the processing of qualitative data, the IRaMuTeQ (Interface de R pour les Analyses Multidimensionnelles de Textes et de Questionnaires), which enables different types of textual data analysis, from simpler analyses, such as basic lexicography (word frequency calculation), to multivariate analyses (descending hierarchical classification) [6].

The textual analysis of this study was divided into 5 stages: the composition of the corpus; the elaboration of command lines; the correction and revision of the corpus; data processing; and Descending Hierarchical Classification (CHD) or the Reinert Method.

The Descending Hierarchical Classification was used to classify text segments (ST) by grouping the corpus vocabularies that present similarity to each other, organizing the analysis of the data into classes and the relationships among them. This classification is achieved through several chi-square tests (x^2^). The description of the main results is presented in the form of a dendrogram showing the partitions and classes [7]. The dendrogram allows for a visualization of the words that obtained the highest percentage of the average frequency between each other and the differences between them. The words considered significant were the words that presented x^2^ greater than 3.84 and *p* < 0.0001 [8].

### 2.3. Ethical Considerations

This project was approved by the Research Ethics Committee of the National Institute of Women, Children and Adolescents Health Fernandes Figueira/FIOCRUZ under opinion 4.102.925 and registered on the Brazil Platform under CAAE: 31997320.5.0000.5269. All participants accepted a Free and Informed Consent Form Online.

## 3. Results

The 173,178 respondents were registered; of these, 18,250 were vaccine-hesitant people, of which 11,248 reported their reasons for hesitation. After reviewing the databases, the number of valid answers was 10,928. Only 320 responses were excluded. The exclusion criterion was to have used the response field for comments that did not correspond to reasons of vaccine hesitancy. Of these, 6182 (56.57%) were female, 5389 (49.31%) were 40 to 59 years old, 6784 (62.08%) were female and 8620 (78.88%) attended higher education. Among the vaccine-hesitant people, there were 2583 (23.64%) who said they did not intend to take the COVID-19 vaccine, a group that showed doubt composed of 6980 (63.87%) individuals and a group that could delay their vaccination due to preference concerns about the type of vaccine offered composed of 1365 (12.49%) individuals.

**Figure 1:**
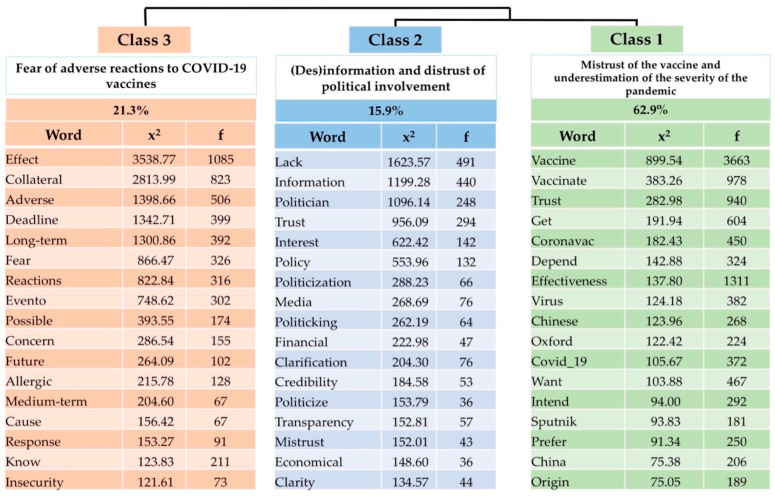
Dendrogram of the Descending Hierarchical Classification. (N = 10,928).

The data processing performed by the IRaMuTeQ software generated 11,262 text segments (ST) of the corpus, and the analysis produced by CHD classified 11,158 of the ST with a utilization of 99.08%, giving rise to three classes (Figure 1). 

### 3.1. Class 1: Mistrust of the Vaccine and Underestimation of the Severity of the Pandemic

This class accounted for 62.9% of the text segments (ST) of the corpus. Class 1, representing the mistrust of the vaccine and underestimation of the severity of the pandemic, showed a significant association for the intention to vaccinate only depending on the vaccine offered (x^2^ = 30.03; *p* < 0.001), which shows that the participants are not totally against vaccination but have doubts and insecurities. 

Class 1 represents the central core of the representations among the group of hesitant individuals. We can interpret this class with great centrality about the effectiveness of the vaccine and its brands available on the market. The word “vaccine” has a co-occurrence of 3663 times. The common sense that permeates the group is marked by uncertainties, mistrust and a certain preference for available immunobiological brands. The fear of the antigen platform that uses RNA, given the label of a little-known method, generates doubts in this group. The ST below expresses such insecurity:

*I don’t feel safe to vaccinate myself with vaccines that use innovative and little-known RNA method. I am afraid that problems may arise in the future.* (ID 09667; Female; 18–39 years; Southeast region; Full higher education; maybe, depends on the vaccine)

*In my layman’s view, there are vaccines that are safer and more effective than others. In addition, I’ve had COVID-19, so I have no reason to vaccinate myself as soon as possible with any vaccine. The ideal is to get vaccinated when possible and with a well-established product.* (ID 01161; Male; 18–39 years; South region; Full higher education; Maybe, depends on the vaccine)

Regarding the brands and nationalities of vaccines, the respondents ratify the insecurity that endorses the common sense of hesitation:

*I’ve had COVID-19 and I haven’t seen data released on vaccination in those who have had the disease, whether there are more risks of adverse effects or not. However, if it is made available to me, I intend to vaccinate myself with any vaccine, except for the Russian vaccine that I have so far doubts whether the efficacy data has been manipulated or not, in view of the authoritarian government of the country.* (ID 03583; Female; 18–39 years; Northeast region; Full high school education; Maybe, depends on the vaccine)

*In the case of Pfizer vaccine, I have doubts about the capacity to properly preserve the vaccine in the structure of Brazilian deposits and vaccination stations.* (ID 01132; Male; 40–59 years; Center-West region; Full higher education; Maybe, depends on the vaccine)

*I would not take China and Russia, as China continues to disclose misinformation and hide the origin of the virus. Russia also works with misinformation advertisements. And I don’t trust in US pharmaceutical agencies.* (ID 02478; Male; 18–39 years; Northeast region; Incomplete Higher education; Maybe, depends on the vaccine)

The citation of the maximum percentage of vaccine effectiveness for decision making in vaccinating was addressed in some statements:

*I’d rather wait for a vaccine with 100% effectiveness if it’s not very time consuming.* (ID 07921; Male; 40–59 years; South region; Full higher education; Maybe, depends on the vaccine)

*The vaccine is not fully developed does not have 100% effectiveness I will not take because I have love to my life and I can’t see any advantage in taking a poorly finished vaccine if you will still have to Use mask.* (ID 05194; Prefer not to answer; 18-39 years; Northeast; Incomplete primary school; Does not intend to vaccinate)

Others highlight the virus as a mutant, a fact that invalidates the effectiveness of COVID vaccines, because a vaccine, in their view, will have no effects on any variants:

*Because I think the virus still suffers several mutations, and I believe that this vaccine will still be modified according to in-depth studies.* (ID 05692; Male; 18–39 years; Southeast; Full Higher Education; Does not intend to vaccinate)

Individuals who have already had the disease create an idea that they are protected, immune to the disease. In addition, they also relativize the lethality of the virus, as if any percentage of deaths were acceptable for a vaccine-preventable disease:

*I’ve had COVID-19, I repeated IgG after 5 months and it’s still present. At the moment, it protects me like the vaccine.* (ID 08570; Male; 60–74 years; Southeast region; Post-Graduate degree; Maybe, depends on the vaccine)

*Because a low lethality virus does not deserve this stress. I don’t see myself in the 5% that will evolve. I see myself in the 87%. And if I have to go, I went, it is part of the history of humanity. I don’t cling to the foot of the bed in fear.* (ID 07167; Female; 40–59 years; Southeast; Full Higher Education; Does not intend to vaccinate)

Some deponents still believed in early treatment for the disease, considering that this treatment strategy using hydroxychloroquine or ivermectin was better than vaccinating to prevent COVID-19. In Brazil, the term “early treatment” was used to designate treatment with hydroxychloroquine, ivermectin and other ineffective drugs for the prevention and treatment of COVID-19.

*Because I believe in early treatment, I have had COVID-19 and have taken ivermectin since May 2019. When I had COVID-19 in December, I had only pain in the body and loss of smell and taste, which I recovered in less than 10 days* (ID 00711; Female; 40–59 years; Northeast; Incomplete higher education; does not intend to vaccinate)

### 3.2. Class 2: (Dis)information and Distrust of Political Involvement 

This class accounted for 15.9% of the ST of the corpus. Males were also significantly associated with this class (x^2^ = 85.11; *p* < 0.0001). The age bracket of 60–74 years presented a statistically significant association with this class (x^2^ = 46.84; *p* < 0.0001).

We highlight representative narratives of the difficulty of identifying a reliable source of information. The words “lack” and “information” with the greatest co-occurrence suggest a context of disinformation and lack of transparency of information essential for decision making in the face of a disease little known at the time:

*Due to hyper saturation of information, the most disconnected, putting humanity in a situation as if the vaccine is to be saved* (ID 09113; Male; Incomplete primary school; Maybe, depends on the vaccine)

*Only the Ministry of Health should inform about vaccination. The media is left out informing data from the Ministry of Health and helping in the information of the programs and not confusing the population.* (ID 00963; Male; 60–74 years; Southeast region; Incomplete Higher education; Maybe, depends on the vaccine)

The political use of COVID-19 vaccination was pointed out by the respondents as a fact that generates doubts and/or insecurity. In this class, lexical analysis demonstrated a lack of credibility in politicians, scientists, public people and institutions now renowned, such as the World Health Organization (WHO):

*The deliberations, especially related to COVID-19, are in the hands of politicians. Scientists and health professionals are hostages of political ambition and economic interests like never before seen in the world. This scares me. It is scaring.* (ID 07458; Female; 40–59 years; North; Post-graduate degree; Does not know)

*They have mixed ideology with politics and science. There is no credibility; the institutions have been destroyed.* (ID 00645; Male; 60–74 years; Southeast; Full Higher Education; Does not know)

### 3.3. Fear of Adverse Reactions to COVID-19 Vaccines 

This class accounted for 21.3% of the ST of the corpus. Class 3, which refers to fear of adverse reactions, was more associated with females (x^2^ = 154.99; *p* < 0.0001). The elderly were also associated with this class (age group 60–74 years: x^2^ = 45.84; *p* < 0.0001), and it was also meaningful in this class of people that when asked about the intention to vaccinate some did not know how to answer (x^2^ = 153.77; *p* < 0.0001)

The perception that there may be concealment in the dissemination of information about adverse events of vaccines by the pharmaceutical industry brings even more insecurity and distrust in the group:

*I don’t trust any of the vaccines, I don’t trust the scientific studies funded by Big Pharma. Extreme adverse events are occurring and the industry is exempting itself from the reactions. I do not intend to put a highly toxic substance in my body.* (ID 06117; Male; 40–59 years; Southeast; Full Higher Education; Does not intend to vaccinate)

The justification of not having sufficient and timely studies for the follow-up of adverse events was cited on a recurring basis:

*Because there are no sufficiently consistent studies clarifying about adverse events, nor precise indications or results in the medium term and long term. For the excess of false information both pro and against the vaccine.* (ID 03220; Female; 40–59 years; South; Post-graduate degree; Does not intend to vaccinate).

*Vaccines without satisfactory efficacy, study does not clarify immunization time, nor short-term and long-term adverse events. Use of new techniques with genetic material of the virus until then not tested in humans.* (ID 02413; Male; 40–59 years; Mid-West; Full Higher Education; Does not intend to vaccinate).

The concern about the effects of a new vaccine with the next generations is also recalled by the respondents:

*There are many questions about the long-term effects of vaccines. I am not an anti-vaccine, my daughters have taken all the mandatory vaccines. But I have doubts, for sure. I have read reports about adverse reactions from some vaccines, diabetes for example, which are quite scary.* (ID 09555; Female; 40–59 years; Mid-West; Full Higher Education; Does not know)

## 4. Discussion

The Social Representations Theory (SRT), conceived by the social psychologist Serge Moscovici, reflects on how the production of plural knowledge constitutes and reinforces the identity of groups, and how it influences their practices and how these practices reconstitute their thoughts. This theory contributes to the understanding of the decision of the research participants not to vaccinate and explains the behavior of those who are undecided. After all, the representations can regulate social behaviors and practices, from what groups understand as “socially structured” behaviors that are anchored in certain beliefs and values [9]. 

Some text segments produced demonstrate a minimization of the severity of COVID-19 itself, with the respondents attributing low lethality to the virus or believing that they themselves had a low risk of serious evolutions, either because they had IgG tests positive for COVID-19, have already had the disease or because they relied on the “early treatment” that consisted of the use of ineffective medications for the treatment and prophylaxis of COVID-19, such as ivermectin or hydroxychloroquine. The minimization of the severity of the pandemic should be understood in the Brazilian context, which was crossed throughout the pandemic period by the discrediting of science and the massive dissemination of disinformation [10]. Speeches by the President of the republic discouraging social isolation, as well as minimizing COVID-19 as a minor disease, which he classified as a “little cold”, circulated in various media [11,12]. The discourse adopted by the Bolsonaro government in favor of preserving the economy, to the detriment of the health, depended on the people continuing to work and consume with free circulation. Even with information on the progress of the pandemic, the approach of communication minimizing the severity of the COVID-19 pandemic, contrary to what the international scientific consensus advocated, brought disproportionate and unfair damage to most Brazilians [13]. The consequences of a political and institutional environment of profound obedience to market fundamentalism, combined with a political and ideological agenda aimed at distorting public policies, led to the dismantling of institutions in the areas of health and education [14]

The question of what can offer greater immunity, vaccine or disease, is still a subject of debate in the country and in the world. However, it is important to note that the vaccine has the benefit of not exposing the individual to wild infection with a potential risk of death and of being a form of collective immunization that does not require the infection of the population, which provides a fertile environment to the emergence of new variants. In addition, a study proves the benefit of hybrid immunity for those who have had COVID-19 [15]. This study showed that the rate of SARS-CoV2 infection among unvaccinated persons who had recovered from infection increased from 10.5 among those who had been infected 4 to 6 months previously to 30.2 among those who had been infected 1 year or more previously. However, the adjusted rates were 3.7 and 11.6 among persons who had received a single dose of vaccine after previous infection among those who had been vaccinated less than 2 months and at least 6 months ago, respectively [15]

The response to COVID-19 has been accompanied by a huge infodemic, promoting rumors and misinformation and even the manipulation of information with dubious origins, mainly by social networks [16]. The infodemic tends to aggravate the pandemic by generating anxiety and emotional overload in the population that often cannot have due access to quality information, affecting their decision-making processes [16].

The representations are the desire of individuals to transform something unfamiliar into the familiar. “Every violation of existing rules, a phenomenon or an extraordinary idea, such as those produced by science or technology, abnormal events that disturb what seems to be normal and stable course of things, all this fascinates us, at the same time alarms us” [17]. The COVID-19 pandemic was a phenomenon that shook the world society and generated fear in the population as a result of a disease caused by a new virus that can have very serious effects on people. The search for answers to its questions brought new knowledge closer to much of the population. What was previously only the agenda for discussion in the areas of health sciences has become a topic widely debated in social networks and in the various media.

In June 2020, the official website of the federal government started to present only the new cases without presenting the cases and accumulated deaths, which was understood as a lack of transparency regarding the severity of the pandemic’s advance in the country [18]. The incentive by the Federal Government to use drugs without effectiveness, such as hydroxycloroquine and vitamins, citing a publication of the American Journal of Medicine, led to an editorial of this newspaper to highlight the severity of this type of misinformation especially when it comes from an official organization [19]. 

Reality, however, overlapped mercilessly. The first case of COVID-19 in Brazil was recorded on 26 February 2020 and 25,460,000 cases and 564,710 deaths had already been recorded by 31 January 2021. The second wave of infection in Manaus at the end of 2020 and early 2021, on the eve of this survey, was associated with the emergence and rapid dissemination of the gamma variant, leading to the collapse of the health system with dramatic reports of people dying of asphyxia in the “Earth’s lungs” due to a lack of oxygen in hospitals [20]. The existence of natural immunity prior to SARS-CoV-2 was not capable of preventing the rapid resurgence of transmission and mortality indicating that the variant was not susceptible to naturally acquired immunity [21]. 

Mistrust in the vaccine due to a perceived politicization of the pandemic appeared in the speech of some vaccine-hesitant Brazilian people and had already been pointed out as a harmful factor to vaccine acceptance [22]. International studies correlate political beliefs and attitudes with vaccines. They show a greater intention not to vaccinate in a portion of the population linked to following a political party or according to votes of past elections [23,24]. This study does not include such specificity, but reports indicate that confidence in the vaccine also crosses the political ideology of respondents in Brazil. 

The representations learned in our research converge with the findings of another Brazilian study [25], which identifies the social representations of the COVID-19 pandemic based in negative values, images of discredit in institutions, rulers and media.

The lack of a national representative figure aligned with the global protocols for the control of and fight against the pandemic has generated insecurity in many Brazilians, leading the Federal Supreme Court to delegate to the states the determination of sanitary measures. It was observed that an epidemiological chaotic scenario was progressively installed, plus government crises, policies, exchanges of ministers and conflicts of information, at the Brazilian Ministry of Health [26]. 

Mistrust of pharmaceutical industry interests also appeared in the speeches. The widespread investment of pharmaceutical industries led some respondents to believe that the profit interest of industries would overlap with public health issues [27]. There was also concern about the short time of vaccine development, which was also a cause of hesitation in an American study [28]. The new manufacturing platforms, structure-based antigen design, computational biology, protein engineering and gene synthesis, have provided the tools to now make vaccines with speed and accuracy [29]. In the case of COVID-19, because it is a pandemic, an acceleration in vaccine production was required [30]. 

The wide dissemination, in the Brazilian media of the progress of studies and the phases of vaccine testing provided a follow-up and interest on the part of the population never before seen. Appropriation even of everyday terms of science, such as antibody, antigen, test phases, efficacy and adverse events, occurred at the same speed as the discoveries of scientists were disclosed, generating fear and distrust. Many fake news stories emerged in this pandemic path, confusing the population and generating in many a concern for the future of the disease [10,31]. A qualitative study, conducted in Canada, between 11 February and 19 May 2021, identified among its findings that the large amount of contradictory and confusing information contributed to restrictions in the general understanding of the safety against COVID-19 vaccines [32].

According to Oliveira [33], “these knowledge or representations serve as a guide for groups and individuals to understand new situations, to integrate themselves into social networks and to know how to guide themselves in the world”. The search for this knowledge did not always come from reliable sources of information. The flow of information was intense every day, which also led people to have access to false information, including through messaging apps without any references [34]. The interference of the Brazilian government also contributed strongly with the apprehension of information about vaccines. These groups not only identified and supported the government’s negative perspective but also adopted and reproduced the same statements. The lack of a Brazilian government guideline at the time left the population anchored in numerous imagery constructions about what would be true, which were fake news, generating a common sense of great insecurity and multiple doubts. 

Another very recurrent reason for the participants of this research to hesitate was the degree of effectiveness and the fact that they did not know the response of vaccines in the medium and long term. Initially, there was no evidence of long-term efficacy and safety due to the urgency of the vaccine development, and there was no way to state whether neutralizing antibodies could be maintained for a long time; however, studies showed that most vaccines had good efficacy and safety [35].

Side effects of COVID-19 vaccines are still under study. However, mild reactions have already been identified after the first or second dose of the COVID-19 vaccine, including pain, redness or swelling at the site of vaccine application, fever, fatigue, headache, muscle pain, nausea, vomiting, itching, chills and joint pain, self-limited reactions. It can rarely cause anaphylactic shock. Some people have no side effects. Severe side effects are extremely unlikely after any vaccination, including the COVID-19 vaccine [36]. In a review study with emphasis on clinical efficacy in randomized trials and post-market surveillance of vaccines, it was shown that extremely rare serious adverse events, such as myocarditis and Guillain–Barré syndrome, are much more likely to occur in natural SARS-CoV-2 infection than after vaccination. Moreover, the degree of clinical protection of COVID-19 vaccines remains an important triumph of modern science [37].

Vaccine hesitancy is complex and context-specific, varying over time, location and vaccines. The vaccination behavior of the individual is implicated in a multifactorial context, with factors such as knowledge, skills, attitudes, confidence and access to information and services, as well as cultural and social issues [38,39]. According to the STR, the ideas and beliefs that enable people to live are represented in specific structures and groups to which they are inserted, such as churches, social movements and families [17]. People’s representations of vaccination were affected by these suspicions regarding the efficacy and safety of vaccines propagated during the pandemic. This feeling was shared among the various groups to which these individuals belong.

Moscovici states that popular science is not the same for any individual and forever [17]. Social representations are not static; they can be modified at the same time as the structures or problems of society confront people. The gradual evolution of vaccines in the country has undergone several events of epidemics and technological development. Brazil grew up in the field of public health prevention and gained international recognition, in view of the blocking of the transmission of the virus responsible for smallpox and the important role in the sector of epidemiological surveillance and immunizations that were milestones in the history of public health policies in the country [40]. Even with an anti-vaccine movement in Brazil, the success and recognition of vaccines by a large part of the Brazilian population shows a common sense among different groups that vaccination contributes to the protection of everyone’s health.

The representations expressed by vaccine-hesitant people show that new forms of access to this group and new communication strategies on vaccines are necessary and should be discussed in the field of health sciences to stimulate another vision and provoke a new knowledge or representation. This means understanding the internal dynamics of these groups, with their representations of the world, and then proposing policies and actions that in fact echo and cause changes in the understanding of the role of immunization in people’s health.

Among the limitations of this study, although it was answered by participants from several regions of the country, the southeast region was the most represented with 62.1% of the vaccine-hesitant people who answered the question about the reasons for vaccine hesitancy. Another limitation was the low proportion of respondents among those with lower education who represent the most hesitant people [3]. However, in the exposition of the participants’ speeches, care was taken for representativeness in the classes of participants of different educational profiles and of different regions.

## 5. Conclusions

This study, in the light of social representations, shows that many individuals may not have been protected during a pandemic that killed millions worldwide for failing to receive quality information identified as reliable. Although the public health guidelines written by the holders of scientific expertise are valid for the majority, there is a portion of society that will require an adjustment of how they are communicated.

The confidence of this share of the population will not be attained as before but in a post-modern way. The scientific expert will need to get closer, understanding the individual as part of the process of choice, promoting constant dialog with society and encouraging the immunization of society not only against diseases but also against fake news and negative actors through the application of critical thinking techniques.

The social knowledge, presented by the representations apprehended in this study, shows the difficulty of discerning the reliability of information and a social imagination full of doubts and uncertainties. It is essential to shed light on the sociological imagination so that gaps filled with false information can be dismantled and confronted with scientific knowledge accessible to the population. 

## Data Availability

Research participants when they agreed to informed consent did not allow open sharing of data.
